# Influence of coal gangue mulching with various thicknesses and particle sizes on soil water characteristics

**DOI:** 10.1038/s41598-021-94806-0

**Published:** 2021-07-28

**Authors:** Xiu-Na Han, Ying Dong, Yu-qing Geng, Na Li, Chao-Ying Zhang

**Affiliations:** School of Forestry, Beijing Forestry University, Beijing, 100083 China

**Keywords:** Environmental sciences, Hydrology

## Abstract

Water availability seriously affects vegetation restoration in arid mining areas, and mulching is an effective way to improve soil water conditions. Coal gangue occupies large swathes of land resources, resulting in ecological fragility and various environmental problems. Despite coal gangue having mineral elements similar to those in soil, its potential function as a mulch for soil water conservation has been unclear. Herein, mulching on the surfaces of soil columns with 30 cm height and 15 cm inner diameter was conducted using coal gangue with four particle size ranges (0–0.5, 0.5–1, 1–2, and 2–4 cm) and four thicknesses (4, 8, 12, and 16 cm) under laboratory conditions to investigate water infiltration and evaporation under different conditions. The cumulative infiltration of the treatments with mulching thicknesses of 4 cm (T1), 8 cm (T2), 12 cm (T3), and 16 cm (T4) was 16.1%, 22.9%, 28.6%, and 41.6% greater than that of the control, respectively. The cumulative evaporation of the treatments with particle size ranges of 0–0.5 cm (P1), 0.5–1 cm (P2), 1–2 cm (P3), and 2–4 cm (P4) was 6.5%, 28.6%, 22.9%, and 18.6% lower than the control, respectively. Overall, to enhance the soil water storage capacity in mining areas, the results suggest that coal gangue mulching with a thickness of 8–16 cm and particle size range of 0.5–2 cm is suitable.

## Introduction

Mulching can prohibit the loss of soil water by wind, reduce soil evaporation, and improve the soil hydrothermal status and ecological activity; moreover, it is an important technique for sustaining soil water storage^1,2^. Based on the mulching effect and cost, the materials generally involve synthetic plastic materials, organic materials derived from agricultural and wood waste, and some specific materials, mainly gravel, sand, rock fragments, and zeolites^3–6^. Among the mulching material, gravel mulching is an indigenous approach employed in agricultural production, and it has been used in the arid regions of many countries^7,8^. Gravel mulching has also been conducted for at least 300 years in the arid region of northwestern China^9^. Several field trials have shown that gravel mulching can intercept and store rainfall^10–12^, increase evaporation resistance and reduce evaporation from the soil surface^13,14^, improve the soil water retention capacity^15^, reduce the accumulation of surface salt^16^, influence the microbial community composition and function^17,18^, increase dry matter production^19,20^, and improve energy-use efficiency and economic benefits^21^. However, the mulching efficiency varies widely, depending on the characteristics of the mulch, including particle size^22^, gravel texture^7^, percent mulch coverage^23^, and gravel mulch color^24^. Yuan et al.^14^ found that the evaporation inhibition of gravel mulch was inversely related to the grain size, while Ma and Li^7^ found that the greater mulch thicknesses, the better the effects on maintaining soil water. However, determining the optimal thickness of the gravel mulch requires further testing. In addition, it is well-known that evaporation and infiltration are the dynamic interaction processes between the surrounding microclimate and water at the surface and within the soil^25^. Water infiltration plays a key role in watershed hydrology. Cerdà^26^ reported variations in infiltration at different rock fragment cover ratios. Infiltration and runoff on fallow land slopes with different gravel sizes and coverages were determined by Guo et al.^27^. Further, Dang et al.^28^ studied the influence of different thicknesses and positions of coal gangue on the soil water infiltration. Studies on water infiltration under different mulching conditions are few compared to studies that have investigated evaporation inhibition.

The northwest region is characterized by rich coal, low rainwater, and high evaporation^29^. Coal gangue, as a type of solid waste discharged during coal mining and coal washing, occupies a large amount of land resources owing to accumulated gangue, which eventually results in ecological fragility, various environmental problems, and potential health risks^30^. To date, coal-gangue reclamation has mainly recovered vegetation on covered soils. The greatest challenges for the ecological reconstruction of mine lands are derived from scarce fertile soil^31,32^. The reconstructed soil, which mainly comprises raw soil and is similar to the soil parent material used in the reclamation of coal gangue dump land, was characterized by a lack of organic matter, weak soil structure, and an elevated salt content^31,33^. Mixing coal gangue with soil to reconstruct degraded soil can influence soil infiltration rates and saturated hydraulic conductivity^34,35^. Further, a physical crust is present at the soil surface because of raindrops splashing and the dispersion of aggregates, which hampers water movement and thus the hydrological cycle^36^. Overall, lower soil quality aggravates low water retention.

Given this background, a method to utilize this material can effectively solve the problem of coal gangue accumulation^37,38^. In general, the contents of toxic elements in coal gangue are higher than that in natural soil^39^. However, the concentration levels of trace elements are below permissible limits in some regions^40^. Furthermore, as coal gangue can also be used as a substitute for soil replacement because of its low transportation cost^41,42^. More critically, granular coal gangue has a morphology similar to that of the gravel currently used and contains mineral elements similar to those in soil^41^. As a result, coal gangue has been considered as a mulching material for increasing water efficiency in arid regions. However, the influence of mulching on water infiltration and evaporation characteristics has not been significantly investigated. In this study, we designed an indoor simulated soil column experiment. We hypothesized that the coal gangue mulching on the surface of soil columns can change the movement of water on the soil surface, thereby affecting the infiltration and evaporation of soil water. Our objective was to test the influence of different thicknesses and particle sizes of coal gangue mulch on soil water infiltration and evaporation and to determine an optimized design for coal gangue mulch.

## Materials and methods

### Experimental materials

The research materials were collected from the gangue dump located in Yangchangwan in Lingwu City, Ningxia Hui Autonomous Region. The region has a dry climate with windy and sandy weather in spring and winter. According to records obtained from the China Meteorological Data Service Center, the mean annual temperature in this area is 9 °C, average annual rainfall is 192.9 mm, and average annual evaporation is 1762.9 mm. Specifically, the land subsidence around the gangue dump was filled with coal gangue. The coal gangue having undergone no weathering was taken from the gangue dump. Meanwhile, near the gangue dump, a soil profile about 500 cm high formed due to the continuous excavation of the excavator. The soil profile included a sandy soil layer with a 100 cm raw soil layer containing high clay content 400 cm under the sandy soil. The test soil sample including both sandy soil and raw soil was collected from the profile. Before the experiment, the test soil was air dried and then passed through a 5 mm sieve. In particular, the chunks of raw soil were crushed using a rubber hammer before sieving. The coal gangue was crushed by using a steel hammer, after which it was mixed fully; then, four particle size groups were divided by using stainless steel sieves: 0–0.5 cm (P1), 0.5–1 cm (P2), 1–2 cm (P3), and 2–4 cm (P4).

Soil columns were established by filling PVC pipes (50 cm height; 15 cm inner diameter) with soil. Six small holes with diameters of 1 cm were evenly drilled at the bottom of the PVC pipe for soil water drainage and ventilation. To prevent the leaking of raw soil particles, filter paper and gauze were placed at the bottom of each PVC pipe. Before the soil columns were filled, petroleum jelly was smeared evenly on the inner walls of the PVC pipes to eliminate the influence of water-dominant flow along the walls. According to the dry bulk density with 1.4 g/cm^3^ and water content, a soil sample equivalent to 1.237 kg of dry soil was weighed and the soil layer of 5 cm height was controlled each time. A compactor was used to compact the test soil to a specified height, and the soil layers were roughened individually to connect the soil pores. Finally, soil columns of 30 cm height were established.

Four coal gangue mulch thicknesses, such as 4 cm (T1), 8 cm (T2), 12 cm (T3), and 16 cm (T4) were applied to the top of the soil surface. For each coal gangue thickness treatment, four particle sizes (P1, P2, P3, and P4) were arranged as a cross-over experiment. In addition, a soil column without mulching was constructed as a control (CK). A total of 17 treatments were implemented, and each treatment was repeated three times in the experiment.

### Experimental methods

To determine the soil water infiltration process, the vertical constant head infiltration method was used^43^. A scaled Marriot bottle with an inner diameter of 11.4 cm and a height of 50 cm was used for the water supply. A water outlet with a valve was attached to the bottom of the Marriot bottle, and a rubber tube inserted into a right-angled glass tube was connected to the outlet. The water supply head was controlled at ~ 3 cm using a right-angled glass tube to reduce the influence of water head changes on the infiltration process. The valve was opened, and the time at which the wet peak passed the soil surface was recorded. During water infiltration, the water surface level in the Marriot bottle was recorded every minute for the first 5 min and then every 5 min until the wet peak reached the bottom of the soil column. The entire infiltration experiment lasted for 60 min.

A preliminary test revealed that the shift in the soil column mass was irregular due to the weather, and soil water evaporation was slow due to the thick mulch. The soil columns were shipped to a room with relatively stable conditions at 25 ± 2 °C to alleviate disturbances caused by the variations in the environment. To keep the amount of lamp energy at the same level, an aluminum sheet cylinder (30 cm height; 15 cm inner diameter) was inserted into each PVC pipe until it gets contacted with the surface of the soil column. As room energy was not sufficient to cause water evaporation in the soil under deep mulching, a 275-W infrared lamp was placed at the upper level of the aluminum sheet cylinder to provide energy^44^. There was a 35 cm distance from the bottom of the lamp to the surface of the soil columns. The evaporation experiment was started after 48 h of water leaching due to soil saturation, as indicated by water leakage from the bottom of the soil column^45^. Soil water evaporation was determined using the weighing method. The soil column mass was weighed at 18:00 every day of the evaporation experiment using an electronic scale until the 15th day. Then, the daily weight loss of the soil column was converted to evaporation in mm according to the area and inner radius of the PVC column^46^. Finally, cumulative soil evaporation was summarized over 15 days.

For comparison with evaporation, the infiltration amount at different observation times was determined according to the water layer depth in the soil column. The value was obtained by converting the reading from the Markov bottle scale. The initial infiltration rate (mm/min) is equal to the initial infiltration amount divided by the initial infiltration period; the initial infiltration period of this test was unified to 5 min. The stable infiltration rate (mm/min) is the infiltration amount that did not change in a unit time. The average infiltration rate (mm/min) = the total infiltration amount after reaching stability/the time to reach stable infiltration^47^.

### Data processing

One-way analysis of variance (ANOVA) was used to analyze the differences between the soil water infiltration characteristics among the different treatments. The mean values were compared using the least-significant difference test at 5% probability. Because four particle size ranges and four mulch thicknesses were used, the impact of coal gangue mulching on the infiltration and evaporation was analyzed via two-way ANOVA (general linear model). Based on a large amount of data obtained from infiltration and evaporation experiments, a hierarchical cluster analysis (HCA) was used to standardize the water infiltration and evaporation parameters of each treatment and then classify the most similar treatments into the same category according to the between-groups linkage method. This allowed the average value of the water characteristic parameters of different categories to be compared visually to determine the optimal treatment category^48^. The measurement interval was the Euclidean distance. The results of the HCA were expressed using a dendrogram. The HCA and all statistical analyses were performed using SPSS version 23.0 (IBM, Armonk, NY, USA). The results of the cumulative infiltration and evaporation were plotted using the Origin 2018 software package (Origin Lab, Northampton, MA, USA).

## Results

### Variations in infiltration

For different particle sizes, the differences in water infiltration characteristics among the different mulch thickness treatments were inconsistent (Table 1). The initial infiltration rate of all treatments was significantly higher than that of the CK, where the thicker the mulch layer, the higher the initial infiltration was. Particularly, under the P4 particle size treatments, the difference in the initial infiltration rate among different thicknesses was significant. The stable infiltration rate of the CK was significantly higher than that of all the mulch treatments. The average infiltration rate of the P1 particle size treatments was not significantly different from that of the CK. For the other particle size treatments, the thicker the layer of mulch, the greater the average infiltration rate was.

**Table 1:** Water infiltration characteristics of the different treatments.

Particle size	Thickness	Treatment	Initial infiltration rate (mm/min)	Stable infiltration rate (mm/min)	Average infiltration rate (mm/min)
P1	0	CK	15.3 ± 0.3d	1.7 ± 0.0a	3.3 ± 0.2a
	T1	T1P1	20.2 ± 0.4c	1.5 ± 0.2b	3.7 ± 0.2a
	T2	T2P1	23.7 ± 0.5b	1.0 ± 0.0d	3.6 ± 0.5a
	T3	T3P1	24.3 ± 0.5b	1.1 ± 0.2c	3.9 ± 0.2a
	T4	T4P1	27.9 ± 0.4a	0.9 ± 0.1e	4.1 ± 0.2a
P2	0	CK	15.3 ± 0.3d	1.7 ± 0.0a	3.3 ± 0.2c
	T1	T1P2	21.4 ± 0.6c	1.5 ± 0.2b	3.9 ± 0.2bc
	T2	T2P2	25.4 ± 0.2b	1.2 ± 0.1c	4.2 ± 0.1b
	T3	T3P2	26.7 ± 0.5ab	1.1 ± 0.1c	4.4 ± 0.3ab
	T4	T4P2	27.8 ± 0.5a	1.1 ± 0.0c	4.9 ± 0.2a
P3	0	CK	15.3 ± 0.3d	1.7 ± 0.0a	3.3 ± 0.2c
	T1	T1P3	19.9 ± 0.3c	1.3 ± 0.0b	3.8 ± 0.2bc
	T2	T2P3	24.8 ± 0.3b	1.2 ± 0.1b	4.2 ± 0.1b
	T3	T3P3	25.7 ± 0.2a	1.2 ± 0.3b	4.4 ± 0.3ab
	T4	T4P3	26.2 ± 0.1a	1. 3 ± 0.2b	4.9 ± 0.2a
P4	0	CK	15.3 ± 0.3e	1.7 ± 0.0a	3.3 ± 0.2c
	T1	T1P4	21.2 ± 0.8d	1.1 ± 0.1bc	3.7 ± 0.3bc
	T2	T2P4	24.8 ± 0.2c	1.2 ± 0.1b	4.1 ± 0.2ab
	T3	T3P4	26.8 ± 0.4b	1.1 ± 0.2c	4.2 ± 0.2ab
	T4	T4P4	31.8 ± 0.3a	1.1 ± 0.1c	4.7 ± 0.2a

P1, P2, P3, and P4 represent the treatments of four different particle sizes of coal gangue, 0–0.5 cm, 0.5–1 cm, 1–2 cm and 2–4 cm, respectively. T1, T2, T3, and T4 denote the mulch thickness of coal gangue is 4 cm, 8 cm, 12 cm and 16 cm, respectively. Values are means ± standard error. Different lowercase letters in the same column indicate significant differences among treatments at certain particle sizes (*p* < 0.05) (a > b > c > d > e).

**Figure 1:**
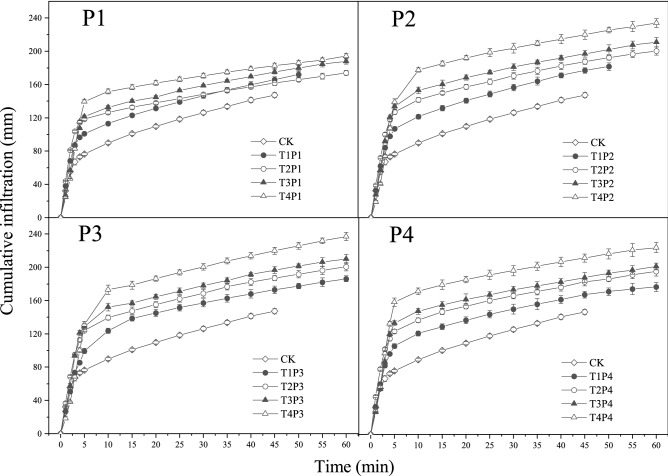
Dynamic variations in cumulative infiltration for different treatments. CK is control treatment without mulched coal gangue. P1, P2, P3, and P4 represent the treatments of four different particle sizes of coal gangue, 0–0.5 cm, 0.5–1 cm, 1–2 cm and 2–4 cm, respectively. T1, T2, T3, and T4 denote the mulch thickness of coal gangue is 4 cm, 8 cm, 12 cm and 16 cm, respectively.

The mulch treatments significantly changed the soil infiltration process in each of the four particle size treatments (Fig. 1). In the initial 0–5 min, cumulative infiltration increased rapidly. After 5 min, cumulative infiltration increased slowly and gradually stabilized. The cumulative infiltration of all mulch treatments was significantly higher than that of the CK. Under the four different particle sizes, cumulative infiltration was greater in the thicker mulch treatments than the thinner mulch treatments. For instance, when the particle size was P4, cumulative infiltration of T4P4 was the largest, followed by T3P4, T2P4, and T1P4.

A two-way ANOVA indicated that both mulch thickness (T) and particle size (P) significantly affected all water infiltration characteristics and cumulative infiltration, and the interaction of mulch thickness and particle size (T × P) significantly affected the initial infiltration rate and stable infiltration rate (Table 2). In particular, the initial infiltration rate increased significantly with the increase in mulch thickness. The effect of mulch thickness on infiltration was more evident than the effects of particle size due to the changes in the F-values. Under the four different mulch thickness treatments, cumulative infiltration was 171.0 mm for T1, 180.0 mm for T2, 189.3 mm for T3, and 208.6 mm for T4. The cumulative infiltration of T1, T2, T3, and T4 was 16.1%, 22.9%, 28.6%, and 41.6% greater than that of the CK, respectively.

**Table 2:** Two-way ANOVA analysis of the effects of mulch thickness and particle size on water infiltration.

Factor	Initial infiltration rate (mm/min)	Stable infiltration rate (mm/min)	Average infiltration rate (mm/min)	Cumulative infiltration (mm)
Thickness (T)	226.49**	51.88**	9.63**	19.43**
T (sig)	d, c, b, a	a, b, bc, c	c, bc, b, a	c, bc, b, a
Particle size (P)	22.10**	20.26**	4.34*	4.41*
P (sig)	c, b, b, a	b, a, a, b	b, a, a, a	b, a, a, a
Interaction (T × P)	7.03**	17.47**	0.34*	0.33*

T (sig) indicates significant differences among the different mulch layer thicknesses of coal gangue (4 cm, 8 cm, 12 cm, and 16 cm). P (sig) indicates significant differences among the different particle sizes of coal gangue (0–0.5 cm, 0.5–1 cm, 1–2 cm, and 2–4 cm). Data are expressed as F-values with the level of significance (*, *p* < 0.05; **, *p* < 0.01). Different lowercase letters indicate significant (*p* < 0.05) differences (a > b > c > d).

### Variations in evaporation

**Figure 2:**
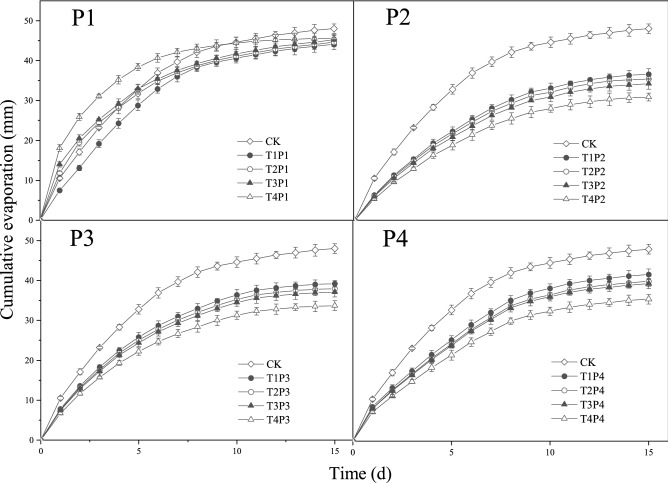
Dynamic variations in cumulative evaporation for different mulch layer thicknesses. CK is control treatment without mulched coal gangue. P1, P2, P3, and P4 represent the treatments of four different particle sizes of coal gangue, 0–0.5 cm, 0.5–1 cm, 1–2 cm and 2–4 cm, respectively. T1, T2, T3, and T4 denote the mulch thickness of coal gangue is 4 cm, 8 cm, 12 cm and 16 cm, respectively.

During the evaporation process, variations in cumulative evaporation were presented according to a logarithmic curve with time (Fig. 2). At the beginning of the evaporation process, all treatments were undergoing massive water loss. As time progressed, the increase in evaporation gradually decreased. At the end of the evaporation process, the cumulative evaporation of all mulch treatments was significantly lower than that of the CK. When the particle size was P1, cumulative evaporation of T1P1 was the lowest. Furthermore, the cumulative evaporation of T2P1, T3P1, and T4P1 were larger than that of the CK until the 4th, 6th, and 10th day, respectively. When the particle size was P2 to P4, cumulative evaporation was lower in the thicker mulch treatments than the thinner mulch treatments. For instance, when the particle size was P2, the cumulative evaporation of T4P2 was the lowest, following by T3P2, T2P2, and T1P2.

A two-way ANOVA indicated that T, P, and their combined effects (T × P) significantly affected cumulative evaporation, whereas particle size exerted the greatest effects on cumulative evaporation due to the changes in the F-values (Supplementary Table S1). Cumulative evaporation decreased significantly with the increase in mulch thickness. In terms of particle size, the cumulative evaporation of the P1 treatment was the largest, and cumulative evaporation increased significantly with the increase in particle size from P2 to P4. Cumulative evaporation was 44.9 mm for P1, 34.2 mm for P2, 37.0 mm for P3, and 39.2 mm for P4. The cumulative evaporation of P1, P2, P3, and P4 was 6.5%, 28.6%, 22.9%, and 18.6% lower than the CK, respectively.

### HCA for soil water characteristics

**Figure 3:**
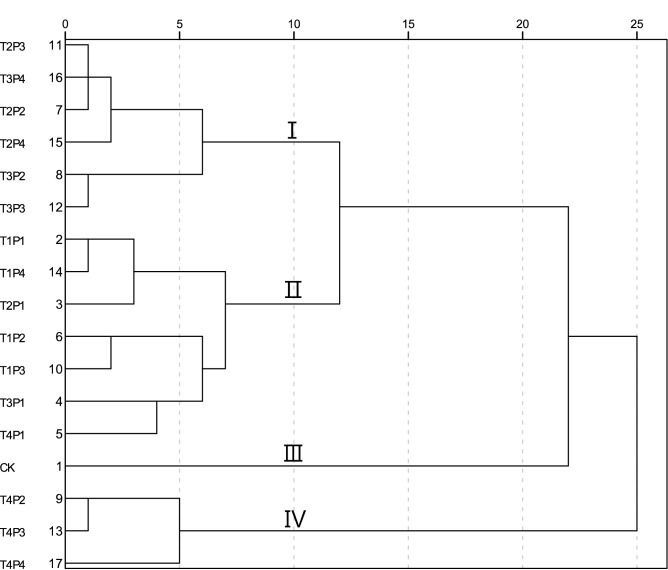
Hierarchical cluster analysis of the water characteristics of the different treatments. The Roman numerals in the figure indicate that all the treatments is divided into four categories according to the Euclidean distance as 10.

All treatments were divided into four categories based on a Euclidean distance of 10 (Fig. 3). Category I included six treatments, involving those with mulch thicknesses T2 and T3 but excluding particle size P1. Category II included seven treatments, involving the treatments with both mulch thickness T1 and particle size P1. Category III only included the CK. Category IV included three treatments, namely T4P2, T4P3, and T4P4.

The soil water infiltration and evaporation parameters of each category were averaged (Table 3). The initial infiltration rate and average infiltration rate of category I and category IV were significantly higher than that of the other two categories. There were no significant differences in the stable infiltration rates among categories I, II, and IV. Furthermore, cumulative infiltration and cumulative evaporation of category IV were significantly higher than that of the other categories. Therefore, the water retention capacity of category IV treatments was the optimal level among the four categories, followed by category I, II, and III.

**Table 3:** Statistical averages of the water infiltration characteristics of the different categories.

Category	Initial infiltration rate (mm/min)	Stable infiltration rate (mm/min)	Average infiltration rate (mm/min)	Cumulative infiltration (mm)	Cumulative evaporation (mm)
I	25.7a	1.2b	4.3b	190.0b	37.4c
II	22.7b	1.2b	3.8c	171.9c	42.4b
III	15.3c	1.7a	3.3d	147.3d	48.0a
IV	28.6a	1.2b	4.8a	217.3a	33.4d

The Roman numerals in the figure indicate the four categories that treatments are divided by hierarchical cluster analysis. Different lowercase letters in the same column indicate significant differences among the different categories (*p* < 0.05) (a > b > c > d).

## Discussion

### Effect of coal gangue mulching on water infiltration

Infiltration is a complex physical process that refers to water penetrating the soil, and can be regulated by soil surface conditions^27^. Previous studies reported that mulching gravel on the soil surface was beneficial to promote water penetration^49,50^. Our findings revealed that the initial infiltration rate and the average infiltration rate increased with the increase in the mulch thickness, whereas the stable infiltration rate decreased (Table 2). This result was partly supported by Zhao et al., who found the initial, stable, and average infiltration rates as well as the cumulative infiltration increased simultaneously with increasing mulch thickness^51^. In regards to the stable infiltration rate, Hu et al.^52^ found that the trend of the stable infiltration rate change with mulching thickness was related to the soil texture. This clearly indicates that the influence of coal gangue mulching thickness on stable infiltration rate needs to be further explored.

Water flow velocities decreased with increasing gravel cover percentages owing to the increase in the infiltration rate^27^. This result was supported by Mandal et al.^49^, who found that the final infiltration rates were 26%, 39%, 62%, and 83% of rainfall and increased with stone mulch coverages from 3 to 65%.

In our study, mulch layer thickness exerted a greater impact on infiltration than particle size of the coal gangue. The reason for the increase in the initial infiltration rate with the increase in coal gangue mulch thickness may be related to the increase in the number of pores and water retention space of the mulch (Table 2).

Previous research has demonstrated that small-sized gravel (2–5 mm) is more effective in controlling surface rainwater runoff than large-sized gravel (40–60 mm), and it can promote increased water entry into the soil surface^18^. In contrast, the smaller the coal gangue grain size, the lower the permeability and the greater the water holding capacity that is observed. Hence, small particle size leads to a decrease in infiltration^36^. This incompatible result is attributed to the range and relativity of the particle sizes. In this study, the impact of particle size on infiltration was also significant. The higher initial infiltration was present for P4, whereas the initial, average, and cumulative infiltration rates were the lowest for P1. This result indicates that mulching with a smaller particle size reduced the infiltration capacity. This is because coal gangue with a fine particle size is more in contact with air and water and can develop narrower capillary pores, enhance water holding capacity, and slow down water infiltration^53^. Conversely, pores in coal gangue with a coarse particle size mainly develop non-capillary pores, and water migrates downwards under the effect of gravity. As a mulching material, the particle size of coal gangue should therefore be greater than P1 to promote water infiltration.

### Effect of coal gangue mulching on water evaporation

In arid areas, mulching can effectively reduce evaporation and improve water content^14,54^. Similar to previous study results, the present results indicate that mulching treatments involve less cumulative evaporation than that in the case of the CK (Fig. 2). Furthermore, the thickness of the mulch layer significantly affected soil water evaporation. However, regarding the optimal mulch thickness, conflicting results were obtained. Doolittle^55^ suggested that no more than 25 mm-thick mulch can best influence the inhibition of evaporation, whereas others have suggested that mulch up to 25 cm can most effectively inhibit evaporation^56^. Contrastingly, some studies have reported that thicker mulch improved the effect on inhibiting evaporation, because the thicker the mulch layer, the greater the evaporation resistance^7,13,57^. Similarly, this study showed that cumulative evaporation significantly decreased with an increase in mulch layer thickness (Table 3).

The particle size of the mulch largely influenced the cumulative evaporation. Previous studies have suggested that cumulative evaporation increases linearly with particle size^24,31^. This is because the capillary pores between the soil and the mulch are discontinuous, thereby blocking upward movement of liquid water^58^. However, when the mulch particles are extremely fine, stronger capillary action results in liquid-phase continuity with the soil^59^. Similar to previous study results, the results of this experiment indicate that the cumulative evaporation increased with an increase in particle size from P2 to P4, whereas the cumulative evaporation for P1 was the largest owing to the mulch being extremely fine to form an effective barrier that inhibited liquid water from moving upward.

Infiltration and evaporation, as the main hydrological processes, influence water distribution of soil at different depth. The regulation of surface infiltration and evaporation is the main water management method in a rain-fed farming system. According to our results, the effects of thickness and particle size on infiltration and evaporation were distinct, and their interactions were evident. Overall, the capacity to hold and transport water related to different coal gangue particle sizes plays a vital role due to the variations in pores among the particle sizes. Furthermore, treatment using a thin layer of fine-particle coal gangue mulch is not recommended because the water storage effect is likely to be weaker than that of a thick layer of large particle mulch. A previous study demonstrated that mulching for water storage should select a heterogeneous particle size^58^. Furthermore, the mixed pebble and sand mulch was more effective in conserving soil water than the pebble or sand mulch used alone^10^. Thus, different coal gangue particle sizes and their applications should be implemented in the future.

In our study, according to the results of HCA (Table 3), category I showed the highest performance in promoting infiltration and inhibiting evaporation, followed by category II. This means that all mulching treatments except T1 and P1 can greatly improve water retention performance. However, since larger particle sizes lead to increased evaporation, P4 should not be prioritized alone in comprehensive evaluations. In addition, we note that while the experiment was conducted at room to investigate the mulching effect, the particular suppressing evaporation effects should be studied in local natural conditions. Further, more experiments under different amounts of light, intensity and spectrum are planned for future work. Finally, although the mulching of coal gangue is cheap and beneficial in utilizing waste, the secondary environmental pollution due to potential trace elements should be assessed before the project starts.

## Conclusions

Owing to the water shortages that seriously limit vegetation restoration in mining areas in northwest China, we selected coal gangue as a mulch and investigated the variations in infiltration and evaporation under different thicknesses and particle sizes. We demonstrated that coal gangue mulching can improve soil water condition by increasing water infiltration and decreasing cumulative evaporation. Based on our study, the coal gangue mulching mode with thickness of 8–16 cm and particle size range of 0.5–2 cm was found to be most effective. Further, the coal gangue mulch technique is suitable for surface soil surrounding trees; for example, coal gangue can be effectively added in planting furrows or around tree disks.

This finding provides a scientific reference for the implementation of the coal gangue mulch technique. However, the research was carried out using soil columns under laboratory conditions, and further research is necessary for the application of this technique to vegetation production. More field trials should be conducted in the future to fully understand how we can meet the water capacity demand of vegetation restoration in mining areas in northwest China.

## Supplementary Information

Supplementary Information.

## Data Availability

The datasets generated and analyzed during the current study are available from the corresponding author on reasonable request.

## References

[1] Kasirajan S, Ngouajio M (2012). Polyethylene and biodegradable mulches for agricultural applications: A review. Agron. Sustain. Dev..

[2] Yimer O (2020). Different mulch material on growth, performance and yield of garlic: A review. Int. J. Food Sci. Agric..

[3] Ozbahce A, Tari AF, Gönülal E, Simsekli N, Padem H (2015). The effect of zeolite applications on yield components and nutrient uptake of common bean under water stress. Arch. Agron. Soil Sci..

[4] Rehman M, Liu J, Johnson AC, Dada TE, Gurr GM (2019). Organic mulches reduce crop attack by sweetpotato weevil (*Cylas formicarius*). Sci. Rep..

[5] Pavlů L (2021). The impact of various mulch types on soil properties controlling water regime of the haplic fluvisol. Soil Till. Res..

[6] Zhang WH, Wei CF, Li Y, Wang GG, Xie DT (2011). Effects of rock fragments on infiltration and evaporation in hilly purple soils of Sichuan Basin, China. Environ. Earth Sci..

[7] Ma Y-J, Li X-Y (2011). Water accumulation in soil by gravel and sand mulches: Influence of textural composition and thickness of mulch layers. J. Arid Environ..

[8] Bonachela S (2020). Effects of gravel mulch on surface energy balance and soil thermal regime in an u-nheated plastic greenhouse. Biosyst. Eng..

[9] Ming W, Yun-wei S (1986). Fruit trees and vegetables for arid and semi-arid areas in north-west China. J. Arid Environ..

[10] Qiu Y, Xie ZK, Wang YJ (2018). Influence of gravel mulch on rainfall interception under simulated rainfall. Soil Water Res..

[11] Kemper WD, Nicks AD, Corey AT (1994). Accumulation of water in soils under gravel and sand mulches. Soil Sci. Soc. Am. J..

[12] Dlamini P, Ukoh IB, van Rensburg LD, du Preez CC (2017). Reduction of evaporation from bare soil using plastic and gravel mulches and assessment of gravel mulch for partitioning evapotranspiration under irrigated canola. Soil Res..

[13] Qiu Y, Xie Z, Wang Y, Ren J, Malhi SS (2014). Influence of gravel mulch stratum thickness and gravel grain size on evaporation resistance. J. Hydrol..

[14] Yuan CP, Lei TW, Mao LL, Liu H, Wu Y (2009). Soil surface evaporation processes under mulches of different sized gravel. CATENA.

[15] Zhao WJ, Cao TH, Li ZL, Su Y, Bao ZW (2020). Spatial variability of the parameters of soil–water characteristic curves in gravel-mulched fields. Water Supply.

[16] Zhao WJ, Cao TH, Li ZL, Sheng J (2019). Comparison of IDW, cokriging and ARMA for predicting spatiotemporal variability of soil salinity in a gravel–sand mulched jujube orchard. Environ. Monit. Assess..

[17] Hao HT (2017). Effects of gravel-sand mulching on soil bacterial community and metabolic capability in the semi-arid Loess Plateau, China. World J. Microbiol. Biotechnol..

[18] Lv WC (2019). Gravel mulching effects on soil physicochemical properties and microbial community composition in the Loess Plateau, northwestern China. Eur. J. Soil Biol..

[19] Lü H (2013). Effect of gravel-sand mulch on soil water and temperature in the semiarid loess region of Northwest China. J. Hydrol. Eng..

[20] Qin Y, Yi SH, Chen JJ, Ren SL, Ding YJ (2015). Effects of gravel on soil and vegetation properties of alpine grassland on the Qinghai–Tibetan Plateau. Ecol. Eng..

[21] Wang DL (2019). Energy input–output, water use efficiency and economics of winter wheat under gravel mulching in Northwest China. Agric. Water Manag..

[22] Xie ZK (2010). Particle-size effects on soil temperature, evaporation, water use efficiency and watermelon yield in fields mulched with gravel and sand in semi-arid Loess Plateau of northwest China. Agric. Water Manag..

[23] Li XY (2005). Influence of pebble size and cover on rainfall interception by gravel mulch. J. Hydrol..

[24] Xie ZK, Wang YJ, Jiang WL, Wei XH (2006). Evaporation and evapotranspiration in a watermelon field mulched with gravel of different sizes in northwest China. Agric. Water Manag..

[25] Mansell M, Rollet F (2009). The effect of surface texture on evaporation, infiltration and storage properties of paved surfaces. Water Sci. Technol..

[26] Cerdà A (2001). Effects of rock fragment cover on soil infiltration, interrill runoff and erosion. Eur. J. Soil Sci..

[27] Guo TL, Wang QJ, Li DQ, Zhuang J (2010). Effect of surface stone cover on sediment and solute transport on the slope of fallow land in the semi-arid loess region of northwestern China. J. Soils Sediments.

[28] Dang HY, Shao MA, Chen HS, Zhou BB (2012). Effect of thickness and location of coal gangue on the process of water infiltration. J. Soil Water Conserv..

[29] Yao QL, Tang CJ, Liu ZH (2020). Analysis of coal and water co-mining in ecologically fragile minin-g areas in western China. Coal Sci. Technol..

[30] Fan J (2013). Pollution of organic compounds and heavy metals in a coal gangue dump of the Gequan Coal Mine, China. Chin. J. Geochem..

[31] Gong YL, Hu ZQ, Mcsweeney K (2020). Reclaiming subsidized land: An evaluation of coal gangue interlayers. Adv. Mater. Sci. Eng..

[32] Lei K, Pan HY, Lin CY (2016). A landscape approach towards ecological restoration and sustainable development of mining areas. Ecol. Eng..

[33] Kuang XY, Cao YG, Luo GB, Huang YH (2019). Responses of *Melilotus officinalis* growth to the composition of different topsoil substitute materials in the reclamation of open-pit mining grassland area in Inner Mongolia. Materials.

[34] Huang YH, Kuang XY, Cao YG, Bai ZK (2018). The soil chemical properties of reclaimed land in an arid grassland dump in an opencast mining area in china. RSC Adv..

[35] Beibei Z, Ming’an S, Mingxia W, Quanjiu W, Horton R (2010). Effects of coal gangue content on water movement and solute transport in a China Loess Plateau soil. Clean: Soil, Air, Water.

[36] Wang J, Li X, Bai Z, Huang L (2017). The effects of coal gangue and fly ash on the hydraulic properties and water content distribution in reconstructed soil profiles of coal-mined land with a high groundwater table. Hydrol. Process..

[37] Taki O, Godwin RJ, Leeds-Harrison PB (2006). The creation of longitudinal cracks in shrinking soils to enhance seedling emergence. Part I. The effect of soil structure. Soil Use Manag..

[38] Wang B (2021). Environmental-friendly coal gangue–biochar composites reclaiming phosphate from water as a slow-release fertilizer. Sci. Total Environ..

[39] Ashfaq M, Heeralal M, Moghal AAB (2020). Characterization studies on coal gangue for sustainable geotechnics. Innov. Infrastruct. Solut..

[40] Tang Q, Li LY, Zhang S, Zheng LG, Miao CH (2018). Characterization of heavy metals in coal gangue-reclaimed soils from a coal mining area. J. Geochem. Explor..

[41] Wang T, Wang Y, Wang J (2008). Research on potential fertilization of coal gangue in the Weibei coalfield, China. Acta Geol. Sin. Engl. Ed..

[42] Du T, Wang DM, Bai YJ, Zhang ZZ (2020). Optimizing the formulation of coal gangue planting substrate using wastes: The sustainability of coal mine ecological restoration. Ecol. Eng..

[43] Cai YK, Li Y, Feng H (2014). Effects of gravel-sand mulching degree and size on soil moisture evaporation. J. Soil Water Conserv..

[44] Zhang CM, Ling L, Lockington D (2015). A physically based surface resistance model for evaporation from bare soils. Water Resour. Res..

[45] Gill BS, Jalota SK (1996). Evaporation from soil in relation to residue rate, mixing depth, soil texture and evaporativity. Soil Technol..

[46] Xiao Q, Zhang HP, Shen YF, Li SQ (2015). Effects of biochar on water infiltration, evaporation and nitrate leaching in semi-arid loess area. Trans. Chin. Soc. Agric. Eng..

[47] Chen LL, Yuan ZY, Shao HB, Wang DX, Mu XM (2014). Effects of thinning intensities on soil infiltration and water storage capacity in a Chinese pine-oak mixed forest. Sci. World J..

[48] Lee HG, Kim HK, Noh HJ, Byun YJ, Chung HM, Kim JI (2020). Source identification and assessment of heavy metal contamination in urban soils based on cluster analysis and multiple pollution indices. J. Soils Sediments.

[49] Mandal UK (2005). Soil infiltration, runoff and sediment yield from a shallow soil with varied stone cover and intensity of rain. Eur. J. Soil Sci..

[50] Poesen J, Ingelmo-Sanchez F, Mucher H (1990). The hydrological response of soil surfaces to rainfall as affected by cover and position of rock fragments in the top layer. Earth Surf. Process. Landf..

[51] Zhao YP, Bai YR, Wang YQ, Yang CX (2017). Investigating and modeling soil infiltration process with gravel mulch on urban green space. J. Northwest A&F Univ. Nat. Sci. Ed..

[52] Hu TF, Wang H, Hu CW, He XJ, Liu HT (2019). Characterization of heavy metals in coal gangue-reclaimed soils from a coal mining area. J. Soil Water Conserv..

[53] Zhang K (2020). Coupled variations of soil temperature and moisture in reclaimed fields filled with coal gangue of different grain size distributions. J. Soils Sediments.

[54] Farzi R, Gholami M, Baninasab B, Gheysari M (2017). Evaluation of different mulch materials for reducing soil surface evaporation in semi-arid region. Soil Use Manag..

[55] Doolittle WE (1998). Innovation and diffusion of sand- and gravel-mulch agriculture in the American southwest: A product of the eruption of sunset crater. Quaternaire.

[56] Tejedor M, Jiménez CC, Díaz F (2003). Use of volcanic mulch to rehabilitate saline-sodic soils. Soil Sci. Soc. Am. J..

[57] Diaz F, Jimenez CC, Tejedor M (2005). Influence of the thickness and grain size of tephra mulch on soil water evaporation. Agric. Water Manag..

[58] Pérez FL (2009). The role of tephra covers on soil moisture conservation at Haleakala's crater (Maui, Hawai'i). CATENA.

[59] Benoit GR, Kirkham D (1963). The effect of soil surface conditions on evaporation of soil water. Soil Sci. Soc. Am. J..

