# Supplementary material for: Influence of coal gangue mulching with various thicknesses and particle sizes on soil water characteristics
Source: Sci Rep. 2021 Jul 28;11:15368. doi: 10.1038/s41598-021-94806-0 (PMC8319200; doi:10.1038/s41598-021-94806-0)
Supplement: Supplementary file 1 — Supplementary Information. [file 41598_2021_94806_MOESM1_ESM.docx]

**Supplementary Table S1** Two-way ANOVA analysis of the effects of thickness and particle size on evaporation

| Factor | Cumulative evaporation  (mm) |
| --- | --- |
| Thickness (T) | 71.12** |
| T (sig) | a, b, c, d |
| Particle size (P) | 495.59** |
| P (sig) | a, d, c, b |
| Interaction (T×P) | 14.85** |

Note: T (sig) indicates significant differences among the different mulching thicknesses of coal gangue (4cm, 8cm, 12cm, and 16cm). P (sig) indicates significant differences among the different particle sizes of coal gangue (0-0.5cm, 0.5-1cm, 1-2cm, and 2-4cm). Data are expressed as F-values with the level of significance (*, p < 0.05; **, p < 0.01). Different lowercase letters indicate significant (p < 0.05) differences (a > b > c > d).
